# Production performance, egg quality, and economic profitability of Isa Brown hens that received *Eucalyptus globulus* leaf powder-based diet

**DOI:** 10.5455/javar.2025.l982

**Published:** 2025-12-25

**Authors:** Aduayi Akue, Lamboni Lare, Edith Aıcha Soara, Essodina Talaki

**Affiliations:** 1 Regional Center of Excellence on Avian Sciences (CERSA), University of Lomé, Lomé, Togo; 2 Togolese Institute of Agricultural Research (ITRA-Togo), Lomé, Togo; 3 Environment and Agricultural Research Institute (INERA), National Center for Scientific and Technological Research (CNRST), Ouagadougou, Burkina Faso; 4 School of Agronomy (ESA), University of Lomé, Lomé, Togo

**Keywords:** *Eucalyptus globulus*, hen, egg quality, profitability

## Abstract

**Objective:**

The *Eucalyptus globulus* leaf powder (EgP) was used in this study to evaluate its effects on the productive performance, physical quality of eggs, and profitability of Isa Brown laying hens.

**Materials and Methods:**

A total of 400 20-week-old Isa Brown laying hens were randomly allocated into 5 groups of 4 replicates each, with 20 hens per pen for 24 weeks. The groups comprised 0% EgP without AGP (negative control, group T-); 0% EgP with the use of AGP (positive control, group T+); 0.25% EgP without AGP (group T1); 0.5% EgP without AGP (group T2); and 1% EgP without AGP (group T3).

**Results:**

The study revealed that the average laying rate was higher in T2 hens with a higher average egg weight, similar to T3. The feed conversion ratio was statistically lower in the EgP and AGP groups than in the negative control group. With storage duration, the eggshell weight was higher, and the Haugh unit significantly decreased less than that of hens treated with EgP compared to the control groups. The gross operating income was positive only for T2 and T3 hens during the 30th to 44th week (phase 2 of laying), and in this phase, the use of *E. globulus* leaf at 0.5% realized a margin of 10.63 F CFA on the production of an egg compared to the antibiotic group.

**Conclusion:**

The inclusion of 0.5% EgP in the diet of hens resulted in the highest level of profitability and egg quality observed throughout the 24-week production period.

## Introduction

Several medicinal plants are used in poultry feed and the treatment of avian diseases [1,2]. *Eucalyptus* has been shown to be effective against bacteria and is a suitable choice for antimicrobial agents [3]. Methanolic and aqueous extracts from *Eucalyptus globulus* leaves showed antimicrobial activity [4,5]. Akue et al. [6] demonstrated that *E. globulus* contains flavonoids, tannins, and phenols with antimicrobial and antioxidant properties. These different properties necessitate the use of *Eucalyptus* in poultry production. Numerous studies have demonstrated the benefits of using *E. globulus* as a feed supplement or as an alternative to antibiotics. Mashayekhi et al. [7] showed that in broiler chickens, the use of *Eucalyptus* as a substitute for antibiotics did not affect the feed intake (FI) during the starter and growth phases, but the body weight gain and feed conversion ratio (FCR) were affected during the finisher and the entire period. *Eucalyptus* supplementation in broiler production did not affect growth performance during the starter and grower phases [8]. Mustafa [9] showed an improvement in weight, body weight gain, and economic benefit of supplementation of broiler feed with *Eucalyptus* leaves. *Eucalyptus globulus* powder did not affect shell quality, albumen, and egg yolk, based on a study conducted by [10]. However, the use of Eucalyptus leaves as a natural feed additive significantly improves eggshell quality and immunocompetence and reduces the number of broken eggs of Japanese quail reared in hot climate conditions [11]. Djenane et al. [12] demonstrated that the Eucalyptus oils tested exhibit anti-*salmonella* activity, making them a promising natural alternative for the preservation of liquid whole eggs. *Eucalyptus globulus* leaf powder (EgP) may be a suitable substitute for antibiotics, as its effects on growth performance, vital organs, and hematological and blood parameters were comparable to or even superior to those of antibiotics [6]. It has been repeatedly demonstrated that the inclusion of Eucalyptus in poultry feed can effectively mitigate the adverse effects of increased egg production [13]. Polyphenols in eucalyptus leaves [6] are valuable resources due to various pharmacological activities that have improved the characteristics of eggs [14].

Aside from the reported benefits of Eucalyptus supplementation in birds’ diets, there are also divergent reports, which are mainly due to varying levels of inclusion and study duration. Mustafa [9] and Ahmed et al. [15] investigated the economic importance of using *E. globulus* leaves in broiler chickens and observed an improvement in the cost of production of supplementation of broiler feed with Eucalyptus leaves. However, there is little information on the economic benefits of the use of Eucalyptus in the feed of poultry, especially in laying hens. It is, therefore, hypothesized that the inclusion of Eucalyptus in the feed of laying hens will improve the production performance and the economics of production. This study aimed to evaluate the production performance, physical quality of eggs, and economic impact of using *E. globulus* leaves in Isa Brown laying hens.

## Materials and Methods

### Ethical approval

The animal care guidelines recommended by the Animal Ethics Committee of the University of Lome in Togo were followed (008/2021/BC-BPA/FDS-UL) [6].

### Experimental design

*Eucalyptus globulus* leaveswere collected in the Ave prefecture, Togo, and were dried and sheltered from light for 72 h with natural ventilation (at a room temperature of 20℃–25°C and relative air humidity of 42%–54%) [6]. Antibiotics were replaced with a powder of *E. globulus* leaves added to the feed at supplementation rates of 0.25%, 0.50%, and 1%. The antibiotic was administered through the drinking water, according to the manufacturer’s dosage for positive control hens [6]. A total of 400 20-week-old Isa Brown laying hens were randomly allocated into 5 treatments, each with 4 replicates of 20 birds, housed in floor pens of identical size (2 × 2 m) in a completely randomized design. The experiment lasted for 24 weeks. The treatments consisted of (1) basic diet (BD) without EgP and antibiotics (Negative control, group T-); (2) BD, without EgP but with antibiotic (positive control, group T+); (3) BD+0.25% EgP without antibiotics (Group T1); (4) BD+0.50% EgP and no antibiotic (group T2); (5)BD+1% EgP without antibiotic (T3). The negative control group received deworming (Levalap), anticoccidial drugs (Anticox), and vitamins (Amin’total); in addition to its products, positive control hens received antibiotics Limoxin ws and doxin 200-ws (The LAPROVET laboratory of France produces anticoccidial, deworming, and Amin’total; INTERCHEMIE werken “De Adelaar” BV Holland produces Limoxin ws and Doxin 200-ws [6]). The products were given according to the manufacturer’s dosage. Table 1 shows the composition and the nutritional values of the different diets by treatment group. The birds were raised on the floor, and feed and water were provided ad libitum [6], and subjected to the same lighting program (16 h of light per day). One hundred and twenty-five eggs in total, 25 eggs taken randomly by treatment, were stored in an ambient environment (room temperature 22°C–26°C, humidity 45%–53%) for 5 weeks. Five (5) eggs were taken per treatment and per week, within 5 weeks, to determine physical qualities. Considering the costs of transport, labor, drying, and powdering, the economic value of *E. globulus* powder is 267 FCFA/kg.

**Table 1.** Composition and characteristics of diets of Isa Brown hens in lay groups.

Ingredients	T-	T+	T1	T2	T3
Maize	58	58	58	58	58
Soya roasted	17	17	17	17	17
Wheat bran	9	9	9	9	9
Fish meal	7	7	7	7	7
Concentrate layer	2	2	2	2	2
Oyster shell	7	7	7	7	7
EgP	0	0	0.25	0.50	1
Nutrient composition					
ME(Kcal/kg)	2,828.41	2,828.41	2,828.86	2,829.32	2,830.24
CP (%)	17.48	17.48	17.50	17.51	17.53
Lysine (%)	0.92	0.92	0.92	0.92	0.92
Methionine	0.36	0.36	0.36	0.36	0.36
Methionine + cystine (%)	0.57	0.57	0.57	0.57	0.57
Calcium (%)	2.53	2.53	2.53	2.53	2.53
Phosphor (%)	0.59	0.59	0.59	0.59	0.59

EgP = *Eucalyptus globulus* leaf powder; ME = Metabolizable Energy and CP = Crude Protein. T-: Groups BD without EgP and no antibiotics, T+: BD without EgP but with antibiotics, T1: BD + 0.25% EgP and without antibiotics, T2: BD 0.5% EgP and without antibiotics, T3: BD+1% EgP without antibiotics.

### Production performance

At the end of each week, the feed given and the uneaten feed were recorded by weight. Lay hens’ eggs were collected daily, counted, and weighed. FI, egg lay rate (ER), and the FCR were calculated for each treatment according to formulas 1, 2, and 3 [16-18].

$$\mathrm{FI}=\frac{\mathrm{Quantity}\;\mathrm{of}\;\mathrm{feed}\;\mathrm{served}-\mathrm{Quantity}\;\mathrm{leftover}}{\mathrm{Number}\;\mathrm{of}\;\mathrm{hens}}\;(1)$$

$$\mathrm{ER}=\frac{\mathrm{Number}\;\mathrm{of}\;\mathrm{egg}\;\mathrm{laid}}{\mathrm{Nnumber}\;\mathrm{of}\;\mathrm{days}\;\mathrm{of}\;\mathrm{lay}\times \mathrm{Number}\;\mathrm{of}\;\mathrm{hens}}\times 100\;(2)$$

$$\mathrm{FCR}=\frac{\mathrm{Quantity}\;\mathrm{of}\;\mathrm{feed}\;\mathrm{consumed}\;\mathrm{by}\;\mathrm{hens}}{\mathrm{Eggs}\;\mathrm{mass}}\times 100\;(3)$$

The mortality (*M*) rate was calculated based on the number of hens and the number of dead hens recorded [19].


$$M=\frac{\mathrm{Number}\;\mathrm{of}\;\mathrm{dead}\;\mathrm{hens}\;\mathrm{per}\;\mathrm{group}}{\mathrm{Tota}l\;n\mathrm{umber}\;\mathrm{of}\;\mathrm{hens}\;\mathrm{per}\;\mathrm{group}}\times 100\;(4)$$

### External and internal eggs’ physical qualities

The different external and internal physical qualities were determined by treatment according to the duration of storage. Egg weight (Ew), shell weight, yolk weight, and albumen weight (Aw) were measured using an electronic scale with a sensitivity of 0.1 gm and a capacity of 5 kg. Large egg diameter (Ld), yolk diameter (Yd), albumen diameter (Ad), egg length (El), yolk height (Yh), and albumen height (Ah) were measured using a caliper ruler. The coloration of the egg yolk was determined using the Roche colorimetric fan (yolk color analyzer) at different levels ranging from light yellow to dark yellow in ascending order of the numbers (1 to 15) corresponding to each color. The shell proportion (PrS), yolk proportion (PrY), albumen proportion (PrA), form index (IndF), yolk index (IndY), albumen index (IndA), and Haugh unit (HU) were calculated using the formulas used by Brasil et al. [19] and Tossou et al. [20].

$$\mathrm{Pr}S=\frac{\mathrm{Sw}}{\mathrm{Ew}}\times 100\;(5)$$

$$\mathrm{Pr}A=\frac{\mathrm{Aw}}{\mathrm{Ew}}\times 100\;(6)$$

$$\mathrm{Pr}Y=\frac{\mathrm{Yw}}{\mathrm{Ew}}\times 100\;(7)$$

$$\mathrm{IndF}=\frac{\mathrm{Ld}}{\mathrm{El}}\;(8)$$

$$\mathrm{IndY}=\frac{\mathrm{Yh}}{\mathrm{Yd}}\;(9)$$

$$\mathrm{IndA}=\frac{\mathrm{Ah}}{\mathrm{Ad}}\;(10)$$

HU =100log(Ah^−1.7^ × Ew0.37 + 7.57) (11)

### Economic parameters

Economic parameters included feed prices, feed costs, production costs, sales prices, and gross operating income (GOI). These different economic indicators were determined by the various phases of production and the treatments applied. The feed price represents the price per kg of feed per treatment. The feed costs reflect the expenditure invested in feed to produce an egg.

The feed cost was calculated through the formula:

Feed costs = Total feed consumed × price of kg feed (12) [21]

The production costs were determined by the formula: [21] is the costs involved in

the production of an egg, concerning the variable and fixed costs. The charges constitute the pullet cost of production at the 20th week according to the proportion for the two laying phases, the cost of the feed ingested by the hens per phase, the cost of medical and health care, the technical depreciation (the life expectancy of the breeding equipment is 3 years and the building is depreciated over 10 years), the labor (2 F CFA per hens per day), and the other charges (electricity, water, transport, communication, transport).

GOI = Products – Cost of production (13) [21].

### Statistical analysis

The statistical analyses were carried out according to the method described by Akue et al. [6]. The collected data were saved in Excel software version 2019 and processed using the R software. The Shapiro-Wilk test was used to check whether the data followed a normal distribution. The differences between mean values were evaluated using the ANOVA test, followed by the Tukey test, with a significance level set at *p* < 0.05. The results were presented as means ± standard error mean [6].

### Phytochemical compositions

The phytochemical composition of *E. globulus* leavesshowed that the powder contained major groups of polyphenols (Table 2) [6].

**Table 2.** Polyphenols’ composition of *E. globulus* leaves.

Parameters	Concentration
Total flavonoids	4.85 μg QE / mg
Tannins condensed totals	30.34 μg CE / mg
Total phenols	165.2 μg GAE/mg

QE: Quercetin equivalent, CE: Catechin equivalent, GAE: Gallic acid equivalent, μg: microgram, mg: milligram.

### Physicochemical parameters

The physicochemical composition of *E. globulus* leaves is shown in Table 3 [6].

**Table 3.** Physicochemical composition of *E. globulus* leaves.

Physico-chemical characteristics	Values
Moisture content (% m/m)	52.60
Total nitrogen content (% m/m)	0.70
Protein content (% m/m)	4.37
Fat content (% m/m)	1.12
Ash content (% m/m)	3.04
Carbohydrate content (% m/m)	38.85
Energy value in kilocalorie (Kcal)	183.04

## Results

### Egg lay rate

**Figure 1.**
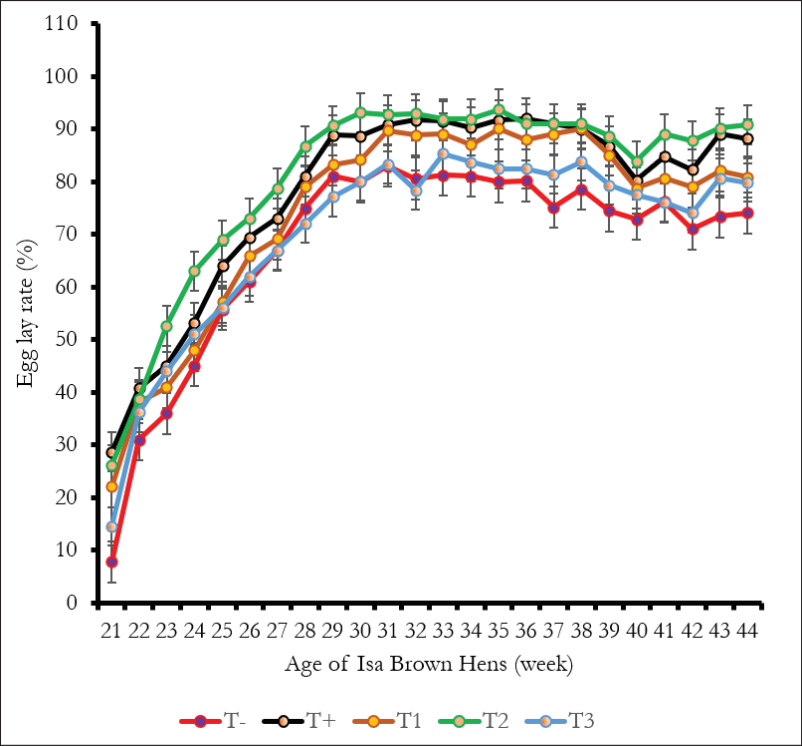
Effect of EgPon the laying rate of Isa Brown hens. T-: Groups fed BD without EgP and no antibiotics, T+: BD without EgP but with antibiotics, T1: BD + 0.25% EgP and without antibiotics, T2: BD 0.5% EgP and without antibiotics, T3: BD+1% EgP without antibiotics.

Figure 1 shows the egg-laying rates of hens by treatment. Generally, the laying curve indicates two phases. The first phase represents the ascending phase, from the 21st to the 29th week, and the second phase covers the period from the 30th to the 44th week. ER varied according to each phase and averaged 51.06% ± 1.90%, 60.43% ± 2.12%, 55.90% ± 1.99%, 64.26% ± 2.09%, and 53.32% ± 1.98%, respectively, for groups T-, T+, T1, T2, and T3 in phase 1. For the second laying phase, the average egg-laying rates were 77.67% ± 1.82%, 88.6% ± 3.75%, 85.80% ± 2.06%, 90.60% ± 3.82%, and 80.57% ± 1.65%, respectively, for the treatment groups. The different egg-laying rates of hens that received antibiotic treatment, and those that received 0.5% EgP, were statistically higher than those of the other group (*p* < 0.05). Group T-recorded the lowest egg-laying rate (*p* < 0.05), followed by the laying hens that received 1% EgP.

### Egg weight

**Figure 2.**
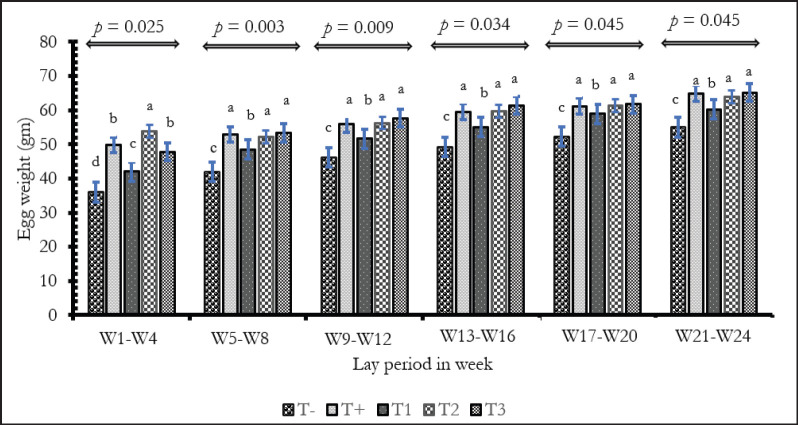
Effect of *E. globulus* leaves on Ew of Isa Brown hens. *p*: plus-value; ^a,b,c^ On the same month, the values assigned different letters are significantly different (*p* < 0.05). T-: Groups fed BD without EgP and no antibiotics, T+: BD without EgP but with antibiotics, T1: BD + 0.25% EgP and without antibiotics, T2: BD+ 0.5% EgP and without antibiotics, T3: BD+1% EgP without antibiotics; W: week.

Figure 2 shows the weight of eggs per treatment over the first 6 months of lay.

The average Ews differed depending on treatment and laying period. From the 21st to the 24th week of lay, the average weight ranged from 36 to 55 gm in the negative control group and from 49.78 to 64.92 gm in the positive control group. The groups received 0.25%, 0.5% and 1% of EgP showed variations from 42.03 to 60.20 gm, 53.86 to 63.9 gm, and 47.82 to 65.11 gm, respectively. The overall average values of Ews over the 24th months of laying were respectively 46.76 ± 2.20 gm; 57.36 ± 1.86 gm; 52.71 ± 2.18 gm; 57.88 ± 1.55 gm; and 57.85 ± 2 gm of groups T-, T+, T1, T2, and T3. The Ews of T+, T2, and T3 were identical and statistically higher than those of T- and T1 groups (*p* < 0.05).

### FI, FCR, and mortality rate

FI, FCR, and mortality rate (%M) for the different groups by phase are presented in Table 4. Hens that received 1% EgP (T3) had statistically lower FI, followed by the T2 and T1 groups in both laying phases (*p* < 0.05). FCR and mortality followed a similar trend; the T-group had the highest values, while the T+, T1, T2, and T3 groups were identical and statistically lower (*p* < 0.05).

**Table 4.** Effect of *E. globulus* leaves on FI, FCR, and mortality rate of Isa Brown hens.

Variable	Treatment						
		T-	T+	T1	T2	T3	*p*
FI (gm/bird/day)	20–29 weeks.	115.18^a^	113.27^b^	115.55^a^	109.36^c^	101.73^d^	0.0236
	30–44 weeks.	118.09^a^	116.27^b^	116.35^b^	105.91^c^	96.18^d^	0.0420
FCR	20–29 weeks.	3.16^a^	2.21^b^	2.32^b^	2.08^b^	1.98^b^	0.0076
	30–44 weeks.	2.43^a^	2.01^b^	2.02^b^	2.04^b^	1.63^b^	0.0055
Mortality rate (%)		12^a^	2^b^	3^b^	3^b^	2^b^	0.0112

^a,b,c,d^ Means on the same line, with different letters are significantly different (*p* < 0.05); T-: Groups with BD without EgP and no antibiotics; T+: BD without EgP but with antibiotics; T1: BD + 0.25% EgP and without antibiotics; T2: BD+ 0.5% EgP and without antibiotics; T3: BD+1% EgP without antibiotics; FI: Feed intake; FCR: Feed conversion ratio; *p*: probability.

### Eggs’ internal and external physical quality

The different physical qualities of eggs stored in the ambient environment are presented per treatment and storage period in Tables 5 and 6.

**Table 5.** Effect of EgP on the external physical quality of Isa Brown eggs stored in the ambient environment.

Duration	Variable	Parameters			
		External physical quality			
		Ld (mm)	El (mm)	PrS (%)	IndF
Week 1	T-	42.77	54.75	11.29^d^	0.80
	T+	42.33	54.71	12.64^c^	0.77
	T1	42.95	54.28	12.39^c^	0.79
	T2	42.5	54.51	13.3^b^	0.78
	T3	42.19	54.1	14.87^a^	0.81
	SEM	0.17	0.59	0.33	0.01
	*p*	0.94	0.77	< 0.001	0.84
Week 2	T-	42.89	54.04	11.58^b^	0.8
	T+	41.57	54.75	10.92^b^	0.79
	T1	42.6	54.69	11.3^b^	0.78
	T2	43.96	55.06	12.31^ab^	0.78
	T3	42.27	53.98	14.71^a^	0.8
	SEM	1.95	0.97	0.48	0.02
	*p*	0.09	0.11	0.003	0.76
Week 3	T-	43	54.45	11.06^b^	0.76
	T+	42.59	53.96	11.1^b^	0.78
	T1	41.91	53.1	12.63^ab^	0.78
	T2	43.03	55.45	12.1^ab^	0.77
	T3	42.28	54.89	14.28^a^	0.77
	SEM	2.16	1.47	0.37	0.05
	*p*	0.66	0.14	0.007	0.08
Week 4	T-	42.99	55.76	12.24^b^	0.78
	T+	42.31	55.86	11.03^b^	0.76
	T1	42.87	55.95	11.6^b^	0.78
	T2	43.33	56.09	10.98^b^	0.78
	T3	43.08	55.88	14.59^a^	0.76
	SEM	0.66	2.14	0.43	0.25
	*p*	0.60	0.51	< 0.001	0.11
Week 5	T-	43.68	55.91	12.18^ab^	0.77
	T+	42.98	56.26	12.28^ab^	0.77
	T1	43.14	55.83	10.27^b^	0.81
	T2	42.88	56.99	11.17^b^	0.76
	T3	43.15	56.37	13.73^a^	0.8
	SEM	0.75	0.63	0.32	0.03
	*p*	0.44	0.11	< 0.001	0.66

^a,b,c,d^ On the same column, the values assigned different letters are significantly different (*p* < 0.05). Ld: Large egg diameter; El: egg length; PrS: Shell Proportion; IndF: Form Index; T-: Groups with BD without EgP and no antibiotics; T+: BD without EgP but with antibiotic; T1: BD + 0.25% EgP and without antibiotic; T2: BD+ 0.5% EgP and without antibiotic; T3: BD+1% EgP without antibiotic; SEM: Standard error of Means ; *p*: probability.

**Table 6.** Effect of *E. globulus* leaves on the internal physical quality of Isa Brown eggs stored in the ambient environment.

Duration	Variable	Parameters								
		Internal physical quality								
		Yd (mm)	Ad (mm)	Yh (mm)	Ah (mm)	PrY (%)	PrA (%)	IndY	IndA	Yc
Week 1	T–	4.61	7.47	18.12	7.98	31.19	57.52^b^	3.93	1.07	4.75
	T+	4.58	7.4	18.12	7.99	31.78	58.53^a^	3.92	1.08	4.75
	T1	4.59	7.5	18.62	7.96	31.57	56.04^c^	4.06	1.06	5.00
	T2	4.56	7.43	19.34	7.85	30.68	56.56^c^	4.02	1.01	5.25
	T3	4.6	7.49	18.68	7.6	29.41	55.72^c^	3.9	1.01	5.50
	SEM	0.42	0.15	0.46	0.19	1.16	0.96	0.09	0.06	0.22
	*p*	0.95	0.07	0.52	0.08	1.06	< 0.001	0.68	0.47	0.14
Week 2	T–	4.49	7.88	17.75	7.53	31.41	57.01	3.95	0.96	4.75
	T+	4.54	8.28	18.77	7.72	29.27	59.81	3.94	0.93	4.50
	T1	4.63	7.62	18.82	7.91	31.38	56.87	4.06	1.07	4.25
	T2	4.3	7.0	19.34	7.78	30.12	57.57	4.5	1.11	4.75
	T3	4.85	7.06	18.68	7.29	30.22	55.07	3.85	1.01	5.00
	SEM	1.12	0.85	2.15	0.91	1.62	2.17	0.92	0.09	0.27
	*p*	0.06	0.10	0.45	0.09	1.62	2.17	0.92	0.09	0.27
Week 3	T–	4.58	7.32	18.85	8.17	30.79	57.0^b^	3.93	1.04	5.25^b^
	T+	4.61	8.22	16.54	8.0	30.41	58.76^a^	3.59	0.83	4.15^b^
	T1	4.59	7.59	16.44	8.22	32.36	55.01^c^	3.66	0.82	8.75^a^
	T2	3.94	7.15	17.85	7.39	30.30	57.54^b^	4.53	1.03	8.75^a^
	T3	4.35	7.13	17.52	7.21	29.95	55.73^c^	4.03	1.12	9.75^a^
	SEM	1.5	0.99	2.03	1.85	2.92	4.29	0.25	0.07	0.48
	*p*	0.24	1.05	0.59	0.24	1.08	0.91	0.76	0.19	< 0.001
Week 4	T–	4.89	8.07	16.47	8.03	31.21	57.55	3.31	0.87	4.25^b^
	T+	4.79	8.32	16.24	8.16	31.62	57.35	3.37	0.74	4.25^b^
	T1	4.09	7.01	17.89	7.64	30.86	58.16	4.37	1.1	9.5^a^
	T2	4.15	7.39	17.52	7.7	29.96	56.46	4.23	1.12	10.25^a^
	T3	4.35	7.13	17.52	7.21	29.95	55.73	4.03	1.12	10.25^a^
	SEM	2.2	1.79	2.6	0.91	1.75	3.78	0.36	0.1	0.53
	*p*	0.13	0.46	0.49	0.21	1.05	1.36	0.63	0.23	< 0.001
Week 5	T–	4.96	7.9	16.67	8.08	30.19	58.93^b^	3.36	0.75	6.75^b^
	T+	4.74	8.28	16.35	8.23	31.34	54.62^c^	4.45	1.05	5.75^b^
	T1	3.87	7.17	16.71	7.31	31.57	54.89^c^	4.46	1.07	9.5^a^
	T2	4.39	6.67	16.95	7.31	31.57	54.89^c^	4.46	1.07	9.5^a^
	SEM	1.87	1.2	2.3	0.85	1.45	1.39	0.44	0.1	0.53
	*p*	0.07	0.14	0.16	0.18	2.02	0.049	0.67	0.08	< 0.001

^a, b, c, d^: In the same column, values assigned different letters are significantly different (*p* < 0.05). Yd: Yolk diameter; Ad: Albumen diameter; Yh: Yolk height; Ah: Albumen height; PrY: Yolk proportion; PrA: Albumen proportion; IndY: Yolk index; IndA: Albumen index; Yc: Yolk color; T–: Groups with BD without EgP and no antibiotics; T+: BD without EgP but with antibiotic; T1: BD + 0.25% EgP and without antibiotic; T2: BD + 0.5% EgP and without antibiotic; T3: BD + 1% EgP without antibiotic; SEM: Standard error of means; *p*: Probability.

### HU of eggs

**Figure 3.**
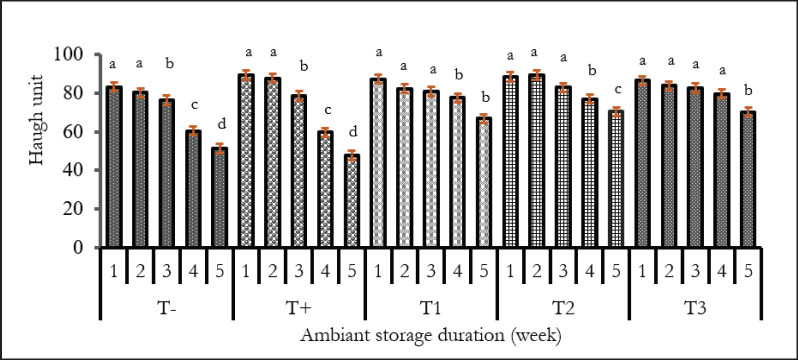
Effect of *E. globulus* leaves on the evolution of the HU of Isa Brown eggs stored in the ambient environment. ^a,b,c^ On the same treatment (T), the values assigned different letters are significantly different (*p* < 0.05). T-: Groups with BD without EgP and no antibiotics, T+: BD without EgP but with antibiotics, T1: BD + 0.25% EgP and without antibiotics, T2: BD+ 0.5% EgP and without antibiotics, T3: BD+1% EgP without antibiotics.

Figure 3 shows the HU of eggs following treatments and storage period. The mean HU values were respectively 83.25 ± 1.57; 89.33 ± 1.74; 87.26 ± 1.01; 88.41 ± 1.88; and 86.59 ± 1.41 of T-, T+, T1, T2, and T3 treatments. At week 5, the mean of HU values was 51.39 ± 1.80; 47.77 ± 1.07; 66.9 ± 1.90; 70.43 ± 1.22; and 70.28 ± 0.75 for the different groups. The highest HU is obtained in the first 3 weeks for all groups. Overall, a decrease in HU was observed with the duration of storage, which became significant during the 3rd week in the eggs of the control group and during the 4th and 5th weeks in the eggs of hens that received EgP(*p* < 0.05).

### Economic parameters

Table 7 presents the results of the economic parameters. The feed price was higher in groups treated with EgP, but no significant difference was observed (*p* > 0.05). In the two egg-laying phases, egg production costs were significantly lower in hens supplemented with EgP at 0.5% and 1% (*p* < 0.05). For the different egg-laying phases, an economic loss was recorded for each first egg-laying phase of each treatment, while benefits were recorded only in the second egg-laying phase, specifically for hens supplemented with 0.5% and 1% EgP.

**Table 7.** Effect of *E. globulus* leaves on Isa Brown hens’ economic parameters.

Period	Variable	Parameters			
		FP	FC	PC	GOI
Laying phase 1	T-	254.32	53.15^a^	84.70^a^	−34.70^b^
	T+	254.32	38.50^d^	84.06^a^	−34.06^b^
	T1	255	43.13^b^	84.95^a^	−34.95^b^
	T2	255.65	35.14^d^	67.30^b^	−17.30^a^
	T3	256.99	39.60^c^	67.21^b^	−17.21^a^
	SEM	-	5.56	2.89	1.56
	*p*	-	0.04	0.01	0.03
Laying phase 2	T-	254.32	59.71^a^	86.25^a^	−16.25^d^
	T+	254.32	44.93^c^	75.81^c^	−5.81^c^
	T1	255	47.15^b^	84.02^b^	−14.02^d^
	T2	255.65	40.75^c^	65.18^d^	4.82^a^
	T3	256.99	41.30^c^	68.15^d^	1.85^b^
	SEM	-	4.992	1.44	2.12
	*p*	-	0.006	0.02	0.04

^a, b, c, d^: In the same column, values assigned different letters are significantly different (*p* < 0.05). FP: Feed prices in F CFA/kg of feed; FC: Feed cost (F CFA/egg); PC: Production cost of an egg in F CFA; GOI: Gross operating income including depreciation; T–: Groups with BD without EgP and no antibiotics; T+: BD without EgP but with antibiotic; T1: BD + 0.25% EgP and without antibiotic; T2: BD + 0.5% EgP and without antibiotic; T3: BD + 1% EgP without antibiotic; SEM: Standard error of means; *p*: Probability.

## Discussion

### Production parameters

Using EgP at a 0.5% concentration improved the egg-laying rate. A similar result was found by [14], who showed that the use of Eucalyptus leaves improves the laying rate of hens. Kaur et al. [10] showed that in Leghorn hens, supplementation of the feed with 0.3% of *E. globulus* leaves improved the laying rate. Likewise, the use of a mixture of medicinal plants, including *E. globulus*, has been shown to increase the laying rate of hens [22]. El-Motaa et al. [13] observed a significant improvement in laying rate when the diets of laying hens were supplemented with Eucalyptus powders. The value found in the first lay phase of the present study for a 0.5% supplementation rate approaches 66.20%, as shown by [10] for a supplementation rate of 0.45% of *E. globulus* during the first 13 weeks of laying.

On the contrary, [11] showed that there was no difference between the egg-laying rates of quails receiving Eucalyptus leaves (at 0.1% and 0.2% rates) and the controls, demonstrating that the use of Eucalyptus leaves has no adverse effects on egg-laying rates. This is confirmed by the present study, which revealed the improvement in the laying rate of Isa Brown hens that were supplemented with 0.5% EgP.

The mean Ews obtained in the present study were 57.88 and 57.85 gm for the hens supplemented with 0.5% and 1% of EgP. These values are similar to the mean of 58.75 gm for Isa Brown eggs for the first 9 months of laying, obtained by [20]. Moreover, the values shown in this study are higher than 48 to 51 gm obtained over the first 3 months of laying by [23]. The average weight of the eggs, therefore, increases with the age of the hens.

FI decreased with increasing EgP levels in the diet, which resulted in an improved FCR. The results of this present study confirm those of [13] and [10], with which the use of Eucalyptus in laying hens decreased FI and improved FCR.

The mortality rate was improved in hens that received *E. globulus* leaves. This result confirms the findings of [6], who found a decrease in the mortality rate of Isa Brown chicks when *E. globulus* leaves were used as an alternative to antibiotic growth promoters.

The improvement of various production parameters can be attributed to the biological activity of the polyphenols contained in EgP, which enhances animal health through their antioxidant, anti-inflammatory, and antibacterial properties [24].

### External physical egg quality

This present study revealed that the Ld was not affected by *E. globulus* leaves. The values in this study align with those reported in [25], which found an average diameter of 4.22 cm for the large Isa Browneggs. The results of the study also corroborate those of [26], which revealed no difference in egg diameter. This finding agrees with the results obtained by [27], who also showed no difference in egg characteristics.

The 1-day El of Isa Brown hens is 51.9 mm [26], which is followed by the values for week-old eggs, and then by the different treatments in this study. There is also a concordance between these results and those published by [28]. According to the latter, hens’ eggs stored for 30 days at room temperature have an average El between 53.22 and 55.34 mm. It also appears from this study that the length of eggs over 5 weeks varies between 53.1 and 56.99 mm, which confirms the results of [29], who showed values of 56.2 and 57.2 mm for the length of hen eggs, which is comparable to the high values of eggs stored over 5 weeks in this study. The results of this study also corroborate those of [30], which indicate that there is no significant difference in the lengths of eggs stored for 0, 2, 4, and 6 weeks.

Regarding the eggshell weight, the result of this study is contrary to the results of [31], who found no difference between the eggshell weight obtained from quails that received 0.1% and 0.2% Eucalyptus leaves, but the Eucalyptus decreased the number of broken eggs. The present study employed high rates of EgP (0.25%, 0.5%, and 1%), which can be attributed to the difference in eggshell weight observed in this study. This study also showed a significant difference in eggshell weight depending on the treatments, which disagrees with the results of [32], who found no storage effect on eggshell weight, and corroborates [33], who reported no effect of storage time on eggshell weight.

Moreover, the results corroborate those of [34] who showed significant differences based on storage time. In this present study, eggshell weight increased as the EgP rate increased in the diet of the hens. This supports the findings that Eucalyptus polyphenols increase eggshell weight [14] since they can interact with proteins and form a protein-polyphenol complex, which strengthens the eggshell [35].

The supplementation of *E. globulus* leaves did not affect the egg shape index. This result confirms those of [10] with the use of *E. globulus* at rates of 0.3%, 0.45% and 0.6%. The same result corroborates those of [13] and those of [29].

### Internal physical egg quality

No differences were observed in the diameter and height of egg albumen and yolk, confirming the results of [11], who showed that there are no significant differences in the height and diameter of egg albumen and yolk of hens treated with Eucalyptus.

Eggs’ IndA and IndY are statistically identical for the different levels of EgP treatment. The values are comparable to those reported by [25], who found values of 0.96 and 4.84 for the egg albumen and IndY, respectively. Adouko et al. [26] detected similar values for the egg Albumen and IndY, which are 1.70 and 4.99. The different indices did not vary with treatments during the 5 weeks of ambient storage. This result confirms [10], who found no significant difference between the egg albumen and IndY for hens supplemented with different levels of *E. globulus*. Similarly, egg Aw varied with the supplement of Eucalyptus leaves at the 1st and 5th week, while the Yw did not change significantly during the 5 weeks at ambient storage.

The yolk color was darker with storage time and with the use of EgP. The colors in the control group became darker from the 5th week, while the groups that received 0.5% and 1% Eucalyptus leaves showed significantly darker colors from the 3rd week. Due to the content of xanthophyll pigments, which caused the darker color with the supplementation [36,37]. This change of color with the storage time has been documented by [30] and [32]. Fathi et al. [31] did not find a significant difference in egg yolk colors in quails supplemented with Eucalyptus leaves at rates below 0.25%. In the control group of this study, the color of the egg yolk may become darker as the storage time of the egg increases due to oxidation of the carotenoids present in the yolk [38]. The yolk color values are comparable to those reported by other authors [10,11,29].

The HU decrease of control hens in high storage time can be explained by the fact that there is liquefaction and a decrease of thick albumen (decomposition of the ovomucin-lysozyme complex) in favor of the liquid albumen, which is accompanied by a migration of the yolk to the highest point of the egg [39] followed by the transition of water from albumen to yolk through the yolk membrane [40]. The *E. globulus* leaves are believed to have prevented egg spoilage and improved egg freshness through their antibacterial properties [12], and with regard to the improvement of HU in hens treated. The results are consistent with those of [32], which showed a decrease in HU of eggs stored at room temperature. The results of this study supported those of [41] and [34]. Indeed, according to different authors, the decrease in HU can reach values of 40, 40.6, and 53.51. In contrast, the present results show a decrease in egg HU below 40 for the control and above 55 for eggs from hens that received Eucalyptus. The difference between this study and those of the authors could be explained by the storage time, which is 5 weeks.

### Economic parameters

The feed price was not significantly higher when *E. globulus* was used at the two laying phases. The increase is related to the use of *E. globulus* leaves as a dietary supplement and, therefore, is added to the BD. The prices of complete feeds from this study are higher than the values of 225 F CFA f for laying feed; the difference between feed prices could be explained by significant fluctuations from 1 year to another, depending on the availability and prices of feed ingredients [42].

The increase in egg production costs for the negative control can be attributed to high FI, despite the price per kilogram of feed being statistically identical. The egg value costs of production in this study were generally lower than those reported by [43], which is 87 F CFA for a complete diet. According to the FAO (2015), the estimated egg production cost in 2010 was 51.72 F CFA, a value lower than the egg production cost obtained in this study. Considering the egg cost produced from the positive control hens, the use of *E. globulus* leaves at 0.5% and 1% saved a profit of 16.76 F CFA and 16.85 F CFA during the first laying phase. In the second phase, the values were 10.63 F CFA and 7.66 F CFA.

*Eucalyptus globulus* leaf supplementation in the diets of Isa Brown laying hens is more profitable from the 10th week of lay in terms of the benefits recorded. The results in general, may be a function of the selling price of a tray of 30 eggs on the market; depending on the size, you can have an egg tray at 1,500 FCFA (eggs from phase 1) or 2,100 F CFA for the tray at the second laying phase; either an egg is 50 F CFA in phase 1 of laying and 70 F CFA in phase 2 according to this study. Our result is consistent with [9], who observed that the use of Eucalyptus in broilers’ diet increases economic profits.

## Conclusion

The results revealed that diet supplementation with EgPat a 0.5% rate improved egg laying rate, Ew, and other production parameters. Furthermore, eggshell weight increases, and the HU does not deteriorate; in fact, both parameters improve with storage time. The use of *E. globulus* leaves is more profitable for Isa Brown laying hens from the 10th week of lay, considering the increased economic profits. The result of this study shows that 0.5% of EgPmay substitute for antibiotics during the laying phase of Isa Brown hens.

## List of abbreviations

Ad, Albumen diameter; AGP, Antibiotics growth promoters; Ah Albumen height; Aw, Albumen weight; BD, Basic diet; EgP, *Eucalyptus globulus* leaf powder; ER, Egg lay rate; El, Egg length; Ew, Egg weight; FC, Feed cost; FCR, Feed conversion ratio; FI, Feed intake; FP, Feed price; GOI, Gross operating income; HU, Haugh unit; IndA, Albumen index; IndF, Form index; IndY, Yolk index; Ld, Large egg diameter; PC, Production costs; PrA, Albumen proportion; PrY, Yolk proportion; PrS, Shell proportion; Sw, Shell weight; Yd, Yolk diameter; Yh, Yolk height; Yw, Yolk weight.
